# The Controversy between Drs. Grant and Hall, and Mr. Newport, Respecting Discoveries in the Nervous System

**Published:** 1838-04

**Authors:** 


THE CONTROVERSY BETWEEN DRS. GRANT AND HALL, AND MR. NEWPORT, RESPECTING DISCOVERIES IN THE NERVOUS SYSTEM OF INSECTS.

In our Number for January, 1837, on the occasion of announcing the award of the royal gold medal of the Royal Society to Mr. Newport, we gave a brief notice of the investigations and discoveries in the anatomy and physiology of insects for which this splendid reward was conferred. In our Number for October, of the same year, we gave a more detailed account of a subsequent paper by the same gentleman, on the Temperature of Insects, and its Connexion with the Functions of Respiration and Circulation. At that time we were so well satisfied of the qualifications of the Council of the Royal Society to decide in such matters, that we never entertained the least doubt as to the propriety of the award of the gold medal, nor, consequently, of the justice of Mr. Newport's claims to that high honour. Very recently, however, the justice of this award, and the legitimacy of these claims, have been disputed by Dr. Grant and Dr. Hall; and, as the questions at issue are not merely important as affecting the credit and fame of physiologists of distinction, and the reputation of the most eminent scientific body in this country, but also as matters of interest in the history of physiology, we purpose taking a review of the whole subject on some future occasion, when the war, which now rages so hotly, has ceased, and left men's minds in a fitter disposition for examining more coolly, and deciding with less risk of being accused of partisanship. In the mean time, we have taken the trouble to put in an English dress the opinion of one, who will be allowed by all to be both an impartial and a competent judge, respecting an important point in the controversy,—the extent of Mr. Newport's discoveries in relation to the previous labours of continental physiologists. Whenever we do enter upon the consideration of this controversy, we shall, of course, entirely pass over the personal matters that have been so improperly and indelicately mixed up with it by Dr. Grant and Dr. Hall; but we cannot help remarking, on the present occasion, that, for our own parts, we would much sooner forego the credit of having made the discoveries contended for, than bear the discredit which must inevitably attach to those who could make attacks of such a nature as every person of well-constituted mind must reprobate.

*Extract from Müller's Jahresbericht über die Fortschritte der Anat. Phys. Wiss. im Jahre,* 1836. (Archiv. Jahrgang, 1835. Heft i. s. 81-4.)

"Among the most distinguished labours in comparative anatomy during the past year, is that of Newport on the nervous system of the *Sphinx Ligustri,* during the last stages of its pupa state; the continuation of former investigations in the Phil. Trans. for 1832. The author gives a very accurate description of the visceral nerve, the cephalic portion of which consists of three ganglia: the first of these lies before the brain, and is connected to it by two roots; the two others lie behind the brain and are also connected with it by roots. From these three ganglia, which give off twigs to the mouth and œsophagus, the main trunk of the visceral nerve arises.

"This investigation has enlarged our knowledge of the facts already adduced by Lyonet, Müller, Brandt, and Straus-Dürkheim; and the author has supplied us with new and important facts in his description of the *nervi accessorii respiratorii.* We have been long acquainted with nerves which arise, in common, from the upper surface of the abdominal cord, between every two ganglia, in the median line, and then divide transversely into two similar nerves. Newport names these nerves, which are principally destined to the muscles of respiration,—*nervi transversi accessorii.* In the larva and pupa of the Sphinx Ligustri, twigs of these nerves unite with *animal nerves* (animalischen nerven) which originate in two roots, one from the abdominal cords, the other from their ganglia. The author has made it appear that these nerves form a particular system, inasmuch as the thread from which they originate is continued over the ganglia and abdominal cord into the median line. The nervi transversi, immediately after their origin, give off, each, a twig which runs over the outer edge of the surface of the nerves and ganglion; and, converging with that of the other side, again unites in the middle line of the abdominal cord, from which the nearest middle thread again arises and comports itself precisely in the same manner in regard to the next ganglion, in order to form the next *nervi transversi*. The last pair of these nerves are spread over the rectum. This system of the nervi transversi is connected anteriorly with the lateral ganglia of the cephalic portion of the visceral nerve: in the gryllus viridissimus the middle thread of this system terminates in a small ganglion; in the Carabi and Gryllotalpa, at each origin of the nervi transversi, there is a small ganglion in the middle thread. The purpose of this system seems to be complex: it is chiefly distributed to the sphincters of the air-pores; it is partly motory; but from its connexion with the visceral nerve and its ganglionic structure in some insects, it would appear to contain also organic fibres. Moreover, since this system is connected with the nerves of the extremities and wings, which come from the animal system or that of the abdominal cord, these connexions are probably for the purpose of intermixing organic branches with the animal system. [This is our interpretation of the interesting observations of Newport.] The termination of these nerves is always in the anal muscles. The author, instigated by an earlier observation of Müller respecting a third column in the abdominal cord of the scorpion, has also again found this nerve here. An important discovery of the author is, that he has found the abdominal cord of insects and crustacea to consist of an anterior and posterior pair of columns. The upper pair of columns has no share in the ganglia of the abdominal cord, which belong exclusively to the under pair of columns. He has made this discovery in examining the nervous system of crustacea and insects hardened in spirits. In this way the analogy of the abdominal cord of insects with the system of the spinal cord of vertebrata becomes much more evident than before, and the discoveries of Bell respecting the motory and sensitive system of spinal nerves, become, at once, applicable to the invertebrata. The columns which want ganglia are the uppermost, and therefore, probably, the motory; the under without ganglia, probably the sensory. If this be the case, the relation is the reverse of that which exists in the vertebrata, in which, as is well known, the motor columns lie undermost. By means of the observations of Newport, the conjecture of Treviranus and E. H. Weber, becomes almost proved, —viz. that the ganglia of the abdominal cord of the articulata, correspond with the ganglia of the spinal nerves (the ganglia of the sensitive roots.) The mixed nerves of the abdominal cord, according to Newport's investigations, originate, in the Astacus marinus, in roots which belong partly to the ganglia and partly to the superior ganglionless columns. In these animals Newport also saw nerves which originate exclusively from the upper columns and not from the ganglia, and are distributed only to muscles. Finally, the author describes the changes of the nervous system during the period of transformation, and here his observations correspond with those of Herold."

---

